# The Challenges of Spirometric Diagnosis of COPD

**DOI:** 10.1155/2023/6991493

**Published:** 2023-09-29

**Authors:** Adriana Maldonado-Franco, Luis F. Giraldo-Cadavid, Eduardo Tuta-Quintero, Alirio R. Bastidas Goyes, Daniel A. Botero-Rosas

**Affiliations:** ^1^Biosciences Doctorate Student, School of Engineering, Universidad de La Sabana, Chía, Colombia; ^2^Departments of Epidemiology and Internal Medicine, School of Medicine, Universidad de La Sabana, Chía, Colombia; ^3^Director of Interventional Pulmonology Service, Fundación Neumológica Colombiana, Bogotá, Colombia; ^4^Candidate for Master's Degree in Epidemiology, Universidad de La Sabana, Chía, Colombia; ^5^School of Medicine, Universidad de La Sabana, Chía, Colombia

## Abstract

Chronic obstructive pulmonary disease (COPD) is one of the top causes of morbidity and mortality worldwide. Although for many years its accurate diagnosis has been a focus of intense research, it is still challenging. Due to its simplicity, portability, and low cost, spirometry has been established as the main tool to detect this condition, but its flawed performance makes it an imperfect COPD diagnosis gold standard. This review aims to provide an up-to-date literature overview of recent studies regarding COPD diagnosis; we seek to identify their limitations and establish perspectives for spirometric diagnosis of COPD in the XXI century by combining deep clinical knowledge of the disease with advanced computer analysis techniques.

## 1. Introduction

**Figure 1:**
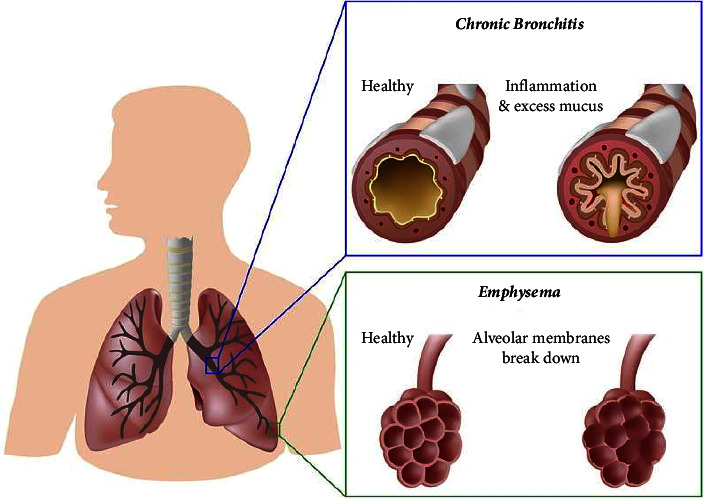
Chronic obstructive pulmonary disease (taken from [1]).

Chronic obstructive pulmonary disease (COPD) is characterized by respiratory symptoms and airflow limitation generated by airway and alveolar alterations. COPD is an umbrella term including chronic bronchitis and emphysema (Figure 1). Functional deviations are triggered by exposure to noxious particles or gases, mainly, smoke from cigarette or biomass combustion. Despite being preventable, it is currently the third leading cause of morbidity and mortality worldwide [2]. In 2019 only, 3.28 million deaths were caused by COPD [3].

**Figure 2:**
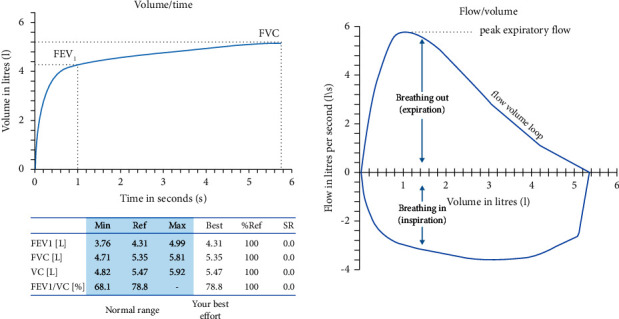
Usual report of a standard spirometry. (a) Flow-volume curve. (b) Volume-time curve. Adapted from [4].

COPD can be diagnosed by several pulmonary function tests (PFTs), but spirometry is the most widely used tool due to its low cost and simplicity.  Figure 2 shows the usual result of a spirometry: two graphs and a summary table of measurements made on the curves included on such graphs [5]. The most important spirometric measurements to detect COPD are the ratio between forced expiratory volume in the first second (FEV1) and forced vital capacity (FVC), both measured during a forced expiration/inspiration manoeuvre after applying a bronchodilator.

**Figure 3:**
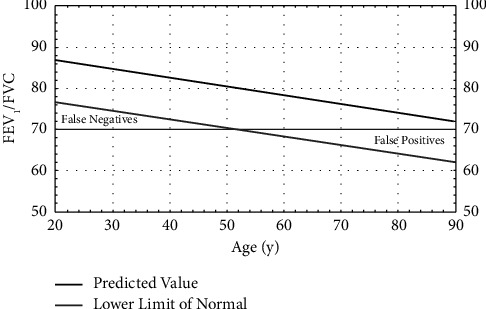
Change of predicted value and LLN of FEV1/FVC with age (taken from [7]).

According to the Global Initiative for Chronic Obstructive Lung Disease (GOLD), if the FEV1/FVC ratio is below 0.7 (70%), the subject is deemed to have COPD. Recommendations from the American Thoracic Society (ATS) and the European Respiratory Society (ERS) include the use of the statistically derived lower limit of normal (LLN) as an alternative to the fixed FEV1/FVC threshold of 0.7 [6], since the LLN includes the effect of normal ageing in the diagnostic process (Figure 3). This graph shows the 70% threshold for reference, the general behaviour of the LLN, and the predicted value according to age, although these parameters also depend on height and sex.

A major effect of having these two case definitions for COPD is a disparity in prevalence estimation. According to Adeloye et al. [8], the global prevalence of COPD in 2019 was 10.3% when using the fixed threshold, and 7.6% according to the LLN definition.

As a tool for establishing the diagnostic performance of a test, a table known as the confusion matrix (also called the diagnostic 2 × 2 table) can be built to classify the diagnosis results (Table 1).

A confusion matrix shows how an index test classifies the subjects in comparison with the truth as defined by a reference test or gold standard. The matrix includes the following four boxes:

- True positives (TP): patients correctly identified as having the disease by the index test
- False positives (FP): patients incorrectly identified as having the disease by the index test
- False negatives (FN): patients incorrectly identified as disease-free by the index test
- True negatives (TN): patients correctly identified as disease-free by the index test

Furthermore, based on the classification shown in this matrix, a few diagnostic metrics can be defined and calculated as follows:

- Accuracy: ability of the test to correctly classify the subject ((TP + TN)/(TP + TN + FP + FN))
- Sensitivity or recall: ability of the test to correctly detect diseased subjects (TP/(TP + FN))
- Specificity: ability of the test to correctly detect disease-free subjects (TN/(FP + TN))
- Positive predictive value: probability that a subject with a positive test result does have the disease (TP/(TP + FP))
- Negative predictive value: probability that a subject with a negative test result does not have the disease (TN/(FN + TN))

The test performance is often assessed on the basis of these metrics, and they are also used to contrast different diagnostic methods.

**Figure 4:**
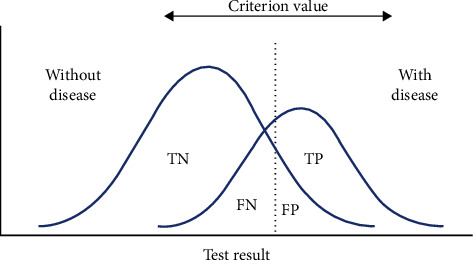
Establishing a threshold for a diagnostic test [9].

Diagnosis is generally based on measuring one or a few variables on the subject. In order to propose a new method, the setting of a threshold value for the discriminatory variable is required. Such variables should discriminate between the population with the disease and the disease-free population. However, it is unusual to find a criterion to perfectly separate both populations since the measured variable in both populations may overlap (Figure 4). Such overlapping means that the threshold value could either favour more false positives or more false negatives, which implies a trade-off between sensitivity and specificity.

Identifying a subject as a false positive or a false negative has important consequences. A false positive COPD diagnosis can lead to a potentially harmful treatment, and it could also hinder the identification and treatment of other potential diseases generating whatever respiratory symptoms in the patient's clinical picture. On the other hand, a false-negative diagnosis could make the patient miss the opportunity to receive timely COPD treatment, which may imply that disease progression may not be managed at an early stage [10]. Bearing this in mind and considering the stage in the diagnostic process, a more sensitive (usually preferred for screening) or a more specific test (better for confirmatory testing) may be used.

To evaluate the diagnostic capabilities of spirometry, repeatability and reproducibility are important parameters to consider. The GOLD, the ATS, and the ERS established certain standards [2, 11] to ensure that a test reaches an appropriate level of quality, and any study involving spirometry should always examine the conditions in which the test was performed [12]. In this case, statistical techniques such as the method agreement analysis [13], intraclass correlation coefficient (ICC) [14], and Bland–Altman plots [15] have proven to be very useful.

Even though the adequate use of spirometry is well described [11], there are some issues regarding its diagnostic accuracy. It is well known that traditional spirometric measures lack sensitivity to detect mild disease. Several reasons may explain such underperformance: first, airflow obstruction diagnosis currently relies on the use of fixed values in the flow-volume curve which are insensitive to small airway disease (where COPD has its early onset) [16]. Secondly, spirometry requires a forced manoeuvre dependent on the patient's effort, which may be variable, and it may be difficult for some patients [17]. This translates into poor reproducibility. And thirdly, any patient with FEV1/FVC ratio below the 95% confidence interval of normal is assumed to be diseased.

This review seeks to provide an overarching perspective of COPD diagnosis, summarise recent COPD diagnostic accuracy studies to understand current hurdles, and identify where there is room for improvement.

### 1.1. Traditional Spirometric Measures

One of the most debatable concepts in COPD diagnosis is whether to use the fixed 0.7 value versus using the LLN as a threshold for the postbronchodilator (post-BD) FEV1/FVC ratio. Efforts have been made to resolve this issue. For instance, Miller et al. [17] compared the clinical characteristics of patients recently diagnosed with COPD by the fixed ratio method and those diagnosed by the lower limit of normal. They found that the fixed ratio identifies more subjects with less respiratory symptoms and more cardiac clinical characteristics.

Furthermore, the following studies have compared the diagnostic accuracy of FEV1/FVC < LLN versus that of FEV1/FVC < 0.7 in different countries. Andreeva et al. [18] compared COPD prevalence in two major cities in Russia using both thresholds as criteria for diagnosing COPD. They included patients with reversible airway obstruction and, if FEV1/FVC < 0.7 is taken as the gold standard, then FEV1/FVC < LLN would have had a sensitivity of 0.69, a specificity of 0.99, and an accuracy of 0.98.

The same comparison was performed in several Canadian cities [19]. If FEV1/FVC < 0.7 is taken as gold standard, then FEV1/FVC < LLN would have achieved an accuracy of 0.94, with relatively low sensitivity (0.64) but perfect specificity [2].

A similar study was carried out in Thailand, where a misidentification prevalence of 5.6% with most subjects in the “underestimated” subgroup was found, meaning that they were identified as false positives when using FEV1/FVC < LLN as the index test and FEV1/FVC < 0.7 as gold standard. The subjects in this “underestimated” group showed significant clinical conditions including chronic respiratory symptoms, so they should not have been considered false positives [20].

Similarly, a study in the Netherlands compared the diagnostic performance of the fixed value versus the LLN with a clinical COPD diagnosis. They found that, while the fixed value was more sensitive than the LLN (0.73 vs. 0.47), it was also less specific (0.95 vs. 0.99) [21].

All the abovementioned studies reported results of spirometric measures after applying a dose of bronchodilators (BD). Nonetheless, some studies have tried to define the impact of not using this medication in spirometric diagnostic accuracy. For instance, Kronborg et al. [10] report that an increase from 64% to 79% in the diagnostic accuracy of FEV1/FVC pre-B2 can be achieved by changing the threshold from 0.7 to 0.66, using FEV1/FVC post-B2 <0.7 as a reference.

On the other hand, completing the forced expiratory manoeuvre can be difficult for some patients for different reasons [22], including the severity of their symptoms or cognitive capacity which impact the FVC measurement quality. Consequently, several studies used spirometric measures at a fixed time point. Particularly, the forced expiratory volume at 6 seconds (FEV6) has been extensively investigated as a replacement for FVC.

For example, in China, Pan et al. [23] determined the diagnostic accuracy of FEV1/FEV6 < 0.73 post-BD vs. FEV1/FVC < 0.7 post-BD, which turned out to have an accuracy of 0.95, a sensitivity of 0.952, and a specificity of 0.945.

Along the same lines, Chung et al. [24] sought to define the best threshold for FEV1/FEV6 pre-B2 to replace FEV1/FVC pre-B2 to detect airway obstruction in a Korean population of 14,978 subjects. A criterion of FEV1/FEV6 < 0.75 pre-B2 achieved a sensitivity of 0.94, a specificity of 0.95, and an overall accuracy of 0.95.

Furthermore, Wang et al. [25] defined the best threshold for FEV1/FEV6 and compared its diagnostic accuracy against FEV1/FVC < 0.70 to detect airway obstruction. This study found that a threshold of 0.75 for FEV1/FEV6 has an accuracy of 0.98, a sensitivity of 0.97, and a specificity of 0.99.

Regarding other spirometric parameters, Ioachimescu et al. [26] proposed an estimation of FVC based on forced expiratory volume at 3 seconds (FEV3) and the diagnostic accuracy of FEV1/FVC3 < LLN, with FEV1/FVC < LLN as the reference test, yielded an accuracy of 0.90, with a sensitivity of 0.94 and a specificity of 0.89.

### 1.2. Nontraditional Spirometric Measures

As mentioned before, traditional spirometric measures are based on specific fixed values which do not seem to take advantage of the wealth of the information the expiratory flow-volume curve has to offer. Some researchers have focused on the description of different measures of the shape of the flow-volume curve.

**Figure 5:**
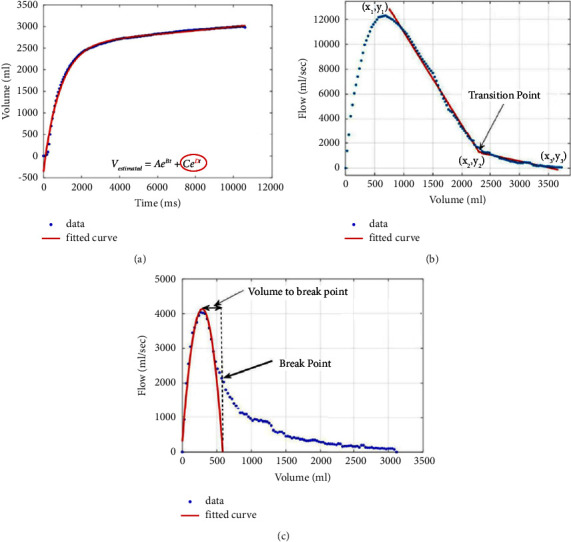
Spirometric measurements proposed by Bhatt et al. in [16], including the *D* parameter.

For instance, Bhatt et al. [16] introduced the *D* parameter (measured in the “volume vs. time” curve) and the transition point and transition distance (measured in the flow-volume curve) and reported its COPD diagnostic accuracy as 0.84, when compared with computed tomography (CT). The measurements proposed in this paper are shown in Figure 5.

**Figure 6:**
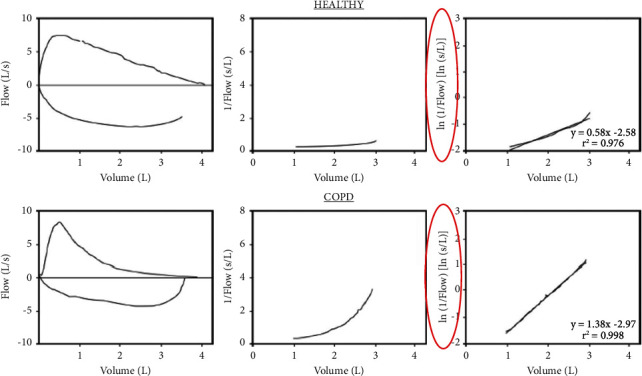
Flow decay in a healthy person vs. in a patient with COPD (taken from [27]).

In addition, Oh et al. [27] proposed the “flow decay,” a measure defined as the slope of volume versus the natural logarithm of the reciprocal of the flow (ln (1/flow)) in midexhalation, to quantify dynamic airway resistance. This measure was found to have an accuracy of 0.94, a sensitivity of 0.95, and a specificity of 0.92 when compared with FEV1/FVC < LLN and plethysmography (Figure 6).

**Figure 7:**
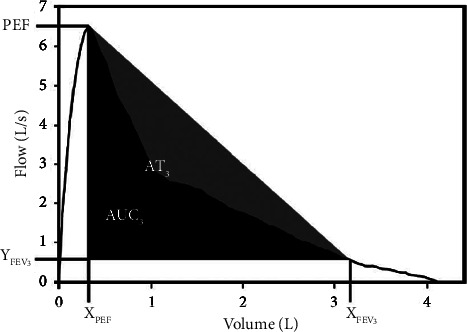
Areas used in [28] to calculate the new parameter AUC_3_/AT_3_.

Li et al. [28] introduced a new parameter, termed the AUC_3_/AT_3_, which is the area under the descending limb of the expiratory flow-volume curve before the end of the first 3 seconds (AUC_3_) divided by the area of the triangle before the end of the first 3 seconds (AT_3_), with an accuracy, sensitivity, and specificity of 0.86, 0.87, and 0.86, respectively, vs. the FEV1/FVC < LLN (Figure 7).

The utility of the area under the expiratory flow-volume curve (AEX) has sparked interest in several researchers due to its apparent ability to detect respiratory abnormalities.

**Figure 8:**
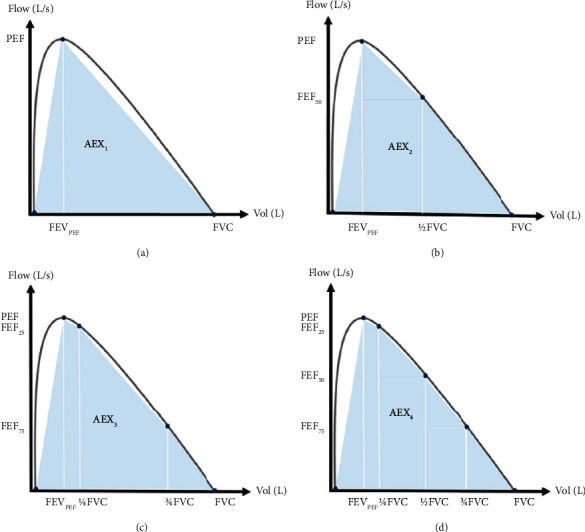
Geometric approximations of AEX proposed in [30].

Several studies [29–31] have been performed regarding the AEX's ability to diagnose respiratory impairment. Ioachimescu et al. [29] found that AEX has a good discriminating capacity between obstruction, restriction, mixed defects, and small airway disease. Later, Ioachimescu and Stoller [30] assessed the diagnostic accuracy and utility of several geometric approximations of AEX based on standard instantaneous flows; they obtained correlations ranging between 0.95 and 0.99 with the actual value of AEX (Figure 8). Ioachimescu and Stoller [31] also evaluated the capability of the square root of one of those approximated values, AEX, to detect and classify bronchodilator responsiveness into five categories: negative, minimal, mild, moderate, and marked, suggesting that this measure could become useful for stratifying dysfunction in obstructive lung disease.

**Figure 9:**
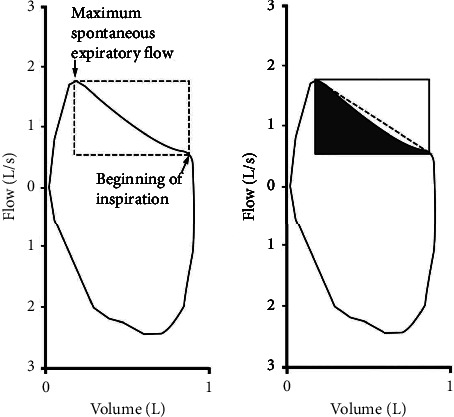
Area under the flow-volume curve as analysed in [32].

Furthermore, the concavity of the expiratory flow-volume curve can also be analysed from the spirometric curves. Nozoe et al. [32] proposed that the concavity/convexity level of the flow-volume curve during spontaneous breathing can be an appropriate replacement for the traditional forced expiratory manoeuvre in older patients. They found that the percent-of-predicted FEV1 had an area under the curve (AUC-ROC) of 0.92, a sensitivity of 0.93, and a specificity of 0.93 as a predictor of the spontaneous expiratory flow-volume curve. In this study, a rectangle defined by the maximum spontaneous expiratory flow and the beginning of the inspiration was calculated, using the area below the curve within the rectangle for diagnosis (Figure 9).

**Figure 10:**
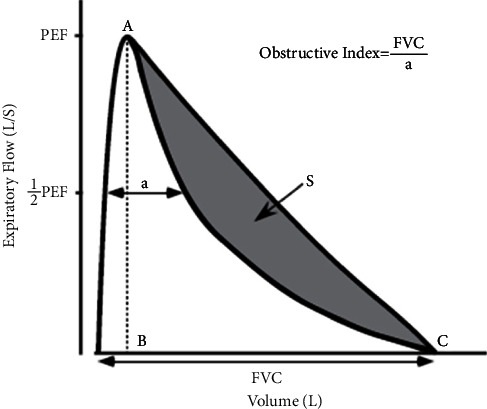
Obstructive index, proposed in [33].

Also, Mochizuki et al. [33] presented a new metric for the maximal expiratory flow-volume curve (MEFV) concavity and proposed a new index, the obstructive index, to quantify the extent of emphysema in COPD, asthma-COPD overlap (ACO), and asthma. This new index, defined as the ratio of forced vital capacity to the difference in volume between the two points where the MEFV curve hits half the value of the peak expiratory flow, had a significant association with the CT measurement of low-attenuation volume (LAV%), which indicates that it could successfully reflect the extent of emphysema (Figure 10).

**Figure 11:**
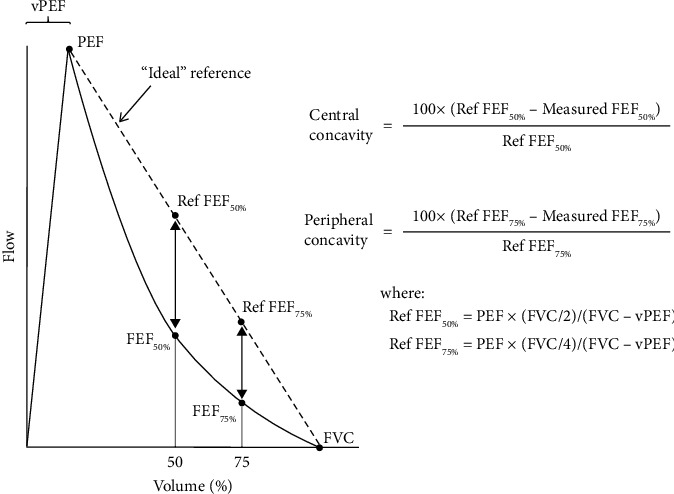
Central and peripheral concavities, as proposed in [34].

Central concavity and peripheral concavity (Figure 11) are other examples of alternative measures, which are calculated based on the forced expiratory flow at 50% and 75% of the forced vital capacity, respectively. Johns et al. [34] found a moderately strong correlation between concavity, FEV1/FVC ratio, and midflow rate. They also found that concavity was more specific for clinical symptoms of COPD.

### 1.3. Machine-Learning Techniques

Lately, artificial intelligence has been used in different fields to improve the performance of diverse systems by trying to emulate the way human intelligence works. Machine learning is a subcategory of artificial intelligence, and it is based on the principle that a computer can learn to perform a task (usually classification or regression) based on examples or experience, and not by being specifically programmed for the task. Deep learning is a machine learning technique that takes advantage of using a vast volume of information to learn.

For example, Das et al. [35] developed a convolutional neural network (CNN) to verify if a flow-volume trace fulfils the ATS/ERS quality control criteria for spirometry. CNN showed an accuracy of 87% for acceptability and 92% for usability in contrast to classifications made by respiratory technicians.

In the case of diagnostic performance for COPD, machine learning has been tested to provide a faster and more accurate diagnostic interpretation of PFTs since it can recognize patterns in high-dimensional feature spaces [36].

Combining their study of AEX with machine learning, Ioachimescu and Stoller [37] proposed the square root of AEX as an alternative spirometric parameter to differentiate between normal, obstructive, restrictive, and mixed patterns. They used machine learning in a model that combined best-split partition and artificial neural networks.

Also, three versions of residual networks were independently trained to perform COPD diagnosis using random subsets of CT scans collected from the PanCan study, which enrolled ex-smokers and current smokers at high risk of lung cancer [38]. These networks were evaluated by using threefold cross-validation experiments. The best performing networks achieved an accuracy of 0.889 (SD 0.017), calculated by the area under the curve (AUC). Moreover, Bodduluri et al. [39] also used CT scans and deep learning to analyse spirometry and they found that ANN and random forests do a better job at phenotyping COPD than the traditional spirometric measurements.

Jafari et al. [40] designed a system to detect normal and abnormal pulmonary functions using spirometry data and multilayer perceptron neural networks (MLPNNs), which classified respiratory patterns into normal, obstructive, restrictive, and mixed patterns, based on the flow-volume curve. This system achieved an accuracy of 0.98, a sensitivity of 0.98, and a specificity of 0.99 across all categories. In a similar study [41], two neural networks were concatenated in such a way that the first classified the sample as normal or abnormal and the second classified abnormal samples into restrictive or obstructive patterns, reporting accuracies, sensitivities, and specificities above 0.90 for all three patterns.

Finally, machine learning has been used not only in diagnosis but also in day-to-day applications to improve the quality of life of COPD patients.

For instance, Swaminathan et al. [42] used a machine learning-based strategy for early detection of COPD exacerbations and subsequent triage. The goal of this study was to identify exacerbations in a timely manner and to evaluate their severity to offer an action plan for the patient. This strategy was compared with the evaluation made by a group of physicians, and it showed good performance in predicting the need for emergency care.

In another study, Cheng et al. [43] proposed a system to classify the lung function based on movement sensors in phones by using support vector machines. This study analysed walking patterns captured by their phone sensors and created a machine-learning model that perfectly classified their pulmonary function into GOLD I/II/III categories.

## 2. Discussion

Ideally, to obtain a COPD diagnosis with certainty, a CT would be the gold-standard. Vimala et al. [44] established a correlation between quantitative and qualitative parameters of high-resolution CT and pulmonary function tests, showing that CT has a key role not only in diagnosis but also in COPD severity definition.

However, CT is not always available, and spirometry is the most used method, at least during the first stages of diagnosis. The most frequently used spirometric measure is the FEV1/FVC ratio with a fixed ratio of 0.7 as the threshold [22]. This value is easy to calculate and remember in a clinical setting and it works reasonably well in the average patient with suspected COPD. Yet, it is well known that this fixed threshold leads to overdiagnosis of older subjects and underdiagnosis of younger subjects because the pulmonary function declines with ageing.

Therefore, when dealing with patients either younger or older than the average, LLN works better. In ideal conditions, the definition of LLN should be obtained by deriving local population-specific equations. However, studies to develop such equations for every population have not been conducted due to logistics and costs. Most studies that use LLN to diagnose COPD or to establish COPD prevalence use known equations (mostly obtained in developed countries, for specific ethnicities) as their reference, which may lead to a decreased diagnostic accuracy. Therefore, an effort should be made globally to develop appropriate LLN equations.

Bhatt and Wood [45] performed a thorough review regarding the controversy around fixed value vs. LLN when dealing with ageing subjects and two important issues were found. First, most studies trying to justify LLN as a better COPD classification tool did not use postbronchodilation, which means that the GOLD recommendations were not fulfilled. Secondly, they found that subjects with FEV1/FVC ratio under 0.7 but over LLN had a higher risk of mortality and hospitalizations due to exacerbations. However, a more recent study [46] found that using FEV1/FVC under 0.7 was not significantly different neither more accurate than other fixed or LLN thresholds in predicting COPD-related hospitalizations or mortality.

In addition, the fixed threshold (0.7) and the LLN for FEV1/FVC have different sensitivities and specificities. This should always be considered in the different stages of COPD diagnosis because they should not be considered interchangeable when used for screening vs. confirmatory testing [47].

Some studies try to exploit data obtained from studies with different goals (e.g., CT data obtained when screening for cancer) or aim at studying spirometric measures without applying bronchodilators. Therefore, these studies do not test bronchodilator response (BDR), which could be an inappropriate practice because, theoretically, not using BDR makes it difficult to differentiate between asthma and COPD and goes against GOLD recommendations for COPD diagnosis.

Interestingly, Janson et al. [48] questioned the use of bronchodilator response in diagnosing COPD due to the limited ability to differentiate asthma from COPD. Also, Fortis et al. [49] studied the impact of bronchodilator response in adverse outcomes measures (such as exacerbations and mortality) and concluded that when BDR is evident in both FEV1 and FVC, the clinical picture is associated with less emphysema, more frequent and severe exacerbations, and lower mortality, suggesting a COPD phenotype with asthma-like features.

Moreover, not all studies check for spirometric repeatability and reproducibility and if they do, they do not always report doing so. Repeatable measurements are critical to guarantee the reliability of the diagnostic test and, when unmet, there is no point in defining the test's diagnostic accuracy. Besides, repeatability is essential for machine-learning models since the models' accuracy will be as good as that of the data used to train them. If there is no quality verification, the achieved models cannot be deemed reliable. Furthermore, machine-learning algorithms would likely benefit from having repeated measurements to learn from.

In addition, since the FEV1/FVC ratio is well known to be an imperfect diagnostic test (whether using fixed or LLN values) [50], it should not be used as a unique criterion to diagnose COPD, nor should it be used as a single gold standard for new diagnostic tests. Whenever possible, all available clinical information should be used to evaluate the diagnostic accuracy of any new method. This is particularly important when training machine-learning models with supervised techniques since the new model will only be as good as the gold standard used to train it.

In fact, some studies suggest that, due to the heterogeneous nature of disease presentation, it is wise to consider its different manifestations beyond spirometry. Lowe et al. [51] segregated current and former smokers into 8 groups, depending on the presence of one or more of the 4 characteristics: exposure (cigarette smoke only), respiratory symptoms (dyspnoea and/or chronic bronchitis), chest CT abnormalities (emphysema, gas trapping, and/or airway wall thickening), and abnormal spirometry, to show how each characteristic contributes to the disease progression and mortality. Adding these nonspirometric characteristics to a machine-learning technique can be easily implemented, which would result in new and perhaps more efficient diagnostic methods.

Recently, the very definition of COPD has been reviewed and a new naming system has been proposed, based on the origin of COPD [52]. This new classification includes 7 definitions: genetic COPD, COPD due to abnormal lung development, COPD due to infections, COPD and asthma, environmental COPD (which has two subcategories: cigarette smoking and biomass and pollution exposure), COPD of unknown causes, and COPD of mixed causes. This study alone may change the way we diagnose COPD considering the different manifestations the disease may have based on its causes.

On a final note, it is remarkable that neural networks are the most frequently used method when applying machine-learning techniques to diagnose COPD. Neural networks are known to be a very powerful tool, but they have a major disadvantage: they are black boxes, meaning that the problem is solved without really understanding the process and the reasoning behind the solution. Perhaps, simpler approaches, which are easier to understand, can be tested to see if their performance is powerful enough to improve the timely diagnosis of COPD.

## 3. Conclusion

COPD is a highly prevalent disease with a significant burden that seriously decreases a patient's quality of life, and its diagnosis remains a challenge despite so many studies being performed on this topic. The heterogeneity of the disease and its multiple origins and presentations make diagnosis a multidimensional problem. Leveraging advanced machine-learning techniques, along with the deep clinical knowledge of the issue, may be the key for tackling the problem and finding more suitable solutions, which should aid in achieving a more efficient diagnosis of COPD.

## Figures and Tables

**Table 1:** Confusion matrix.

		Reference test	
		Disease is present	Disease is not present
Index test	Positive	True positive	False positive
	Negative	False negative	True negative

## Data Availability

The data used to support the findings of the study are in records from the research center.

## Abbreviations

COPD: Chronic obstructive pulmonary disease
PFTs: Pulmonary function tests
FEV1: Forced expiratory volume in the first second
FVC: Forced vital capacity
GOLD: Global Initiative for Chronic Obstructive Lung Disease
ATS: American Thoracic Society
LLN: Lower limit of normal
TP: True positives
FP: False positives
FN: False negatives
TN: True negatives
ICC: Intraclass correlation coefficient
FEV6: Forced expiratory volume at 6 seconds
FEV3: Forced expiratory volume at 3 seconds
CT: Computed tomography
AUC_3_: Area under the descending limb of the expiratory flow-volume curve before the end of the first 3 seconds
AT_3_: Area of the triangle before the end of the first 3 seconds
AEX: Expiratory flow-volume curve
MEFV: Maximal expiratory flow-volume curve
ACO: Asthma-COPD overlap
CNN: Convolutional neural network
AUC: Area under the curve
MLPNNs: Multilayer perceptron neural networks
BDR: Bronchodilator response.
